# A minimally invasive, fast on/off “odorgenetic” method to manipulate physiology

**DOI:** 10.1093/procel/pwae072

**Published:** 2024-12-31

**Authors:** Yanqiong Wu, Xueqin Xu, Shanchun Su, Zeyong Yang, Xincai Hao, Wei Lu, Jianghong He, Juntao Hu, Xiaohui Li, Hong Yu, Xiuqin Yu, Yangqiao Xiao, Shuangshuang Lu, Linhan Wang, Wei Tian, Hongbing Xiang, Gang Cao, Wen Jun Tu, Changbin Ke

**Affiliations:** Institute of Anesthesiology & Pain (IAP), Department of Anesthesiology and Neurosurgery, Taihe Hospital, College of Pharmacy, Hubei University of Medicine, Shiyan 442000, China; Department of Anesthesiology and Pain Medicine, Hubei Key Laboratory of Geriatric Anesthesia and Perioperative Brain Health, Wuhan Clinical Research Center for Geriatric Anesthesia, Tongji Hospital, Tongji Medical College, Huazhong University of Science and Technology, Wuhan 430070, China; Institute of Anesthesiology & Pain (IAP), Department of Anesthesiology and Neurosurgery, Taihe Hospital, College of Pharmacy, Hubei University of Medicine, Shiyan 442000, China; Institute of Anesthesiology & Pain (IAP), Department of Anesthesiology and Neurosurgery, Taihe Hospital, College of Pharmacy, Hubei University of Medicine, Shiyan 442000, China; Department of Anesthesiology, International Peace Maternity and Child Health Hospital, Shanghai JiaoTong University School of Medicine, Shanghai 200030, China; Institute of Anesthesiology & Pain (IAP), Department of Anesthesiology and Neurosurgery, Taihe Hospital, College of Pharmacy, Hubei University of Medicine, Shiyan 442000, China; Institute of Anesthesiology & Pain (IAP), Department of Anesthesiology and Neurosurgery, Taihe Hospital, College of Pharmacy, Hubei University of Medicine, Shiyan 442000, China; Department of Neurosurgery, Beijing Tiantan Hospital, Capital Medical University, Beijing 100070, China; Institute of Anesthesiology & Pain (IAP), Department of Anesthesiology and Neurosurgery, Taihe Hospital, College of Pharmacy, Hubei University of Medicine, Shiyan 442000, China; Institute of Anesthesiology & Pain (IAP), Department of Anesthesiology and Neurosurgery, Taihe Hospital, College of Pharmacy, Hubei University of Medicine, Shiyan 442000, China; Institute of Anesthesiology & Pain (IAP), Department of Anesthesiology and Neurosurgery, Taihe Hospital, College of Pharmacy, Hubei University of Medicine, Shiyan 442000, China; Institute of Anesthesiology & Pain (IAP), Department of Anesthesiology and Neurosurgery, Taihe Hospital, College of Pharmacy, Hubei University of Medicine, Shiyan 442000, China; Institute of Anesthesiology & Pain (IAP), Department of Anesthesiology and Neurosurgery, Taihe Hospital, College of Pharmacy, Hubei University of Medicine, Shiyan 442000, China; Institute of Anesthesiology & Pain (IAP), Department of Anesthesiology and Neurosurgery, Taihe Hospital, College of Pharmacy, Hubei University of Medicine, Shiyan 442000, China; Institute of Anesthesiology & Pain (IAP), Department of Anesthesiology and Neurosurgery, Taihe Hospital, College of Pharmacy, Hubei University of Medicine, Shiyan 442000, China; Institute of Anesthesiology & Pain (IAP), Department of Anesthesiology and Neurosurgery, Taihe Hospital, College of Pharmacy, Hubei University of Medicine, Shiyan 442000, China; Department of Anesthesiology and Pain Medicine, Hubei Key Laboratory of Geriatric Anesthesia and Perioperative Brain Health, Wuhan Clinical Research Center for Geriatric Anesthesia, Tongji Hospital, Tongji Medical College, Huazhong University of Science and Technology, Wuhan 430070, China; State Key Laboratory of Agricultural Microbiology, College of Veterinary Medicine/College of Biomedical Medicine and Health, Huazhong Agricultural University, Wuhan 430070, China; Department of Neurosurgery, Beijing Tiantan Hospital, Capital Medical University, Beijing 100070, China; Institute of Anesthesiology & Pain (IAP), Department of Anesthesiology and Neurosurgery, Taihe Hospital, College of Pharmacy, Hubei University of Medicine, Shiyan 442000, China

**Dear Editor,**

Developing a rapid, controllable method for manipulating physiological functions has significant potential for clinical therapeutics and basic research. Optogenetics has provided optical control of neuronal activity at the millisecond time scale (Bernstein and Boyden, 2011; Carter et al., 2010; Madisen et al., 2012). However, this approach requires direct optical access to brain tissue, which is difficult because blue light does not readily penetrate whole organisms; light must be delivered using costly specialized equipment such as custom blue light sources with fiber optics or two-photon illumination systems (Becnel et al., 2013). Additionally, given the need for invasive equipment implantation and sufficient power (Matveev et al., 2015), it is difficult to apply optogenetics for disease treatment in real clinical practice.

Chemogenetic designer receptors exclusively activated by designer drugs (DREADDs) (Urban and Roth, 2015) are a powerful approach for remote and transient manipulation of cellular activity with no need for specialized equipment (Avaliani et al., 2016; Gomez et al., 2017). A recent study showed that metabolically derived clozapine arising from systemic clozapine N-oxide (CNO) administration is indeed the *in vivo* actuator of DREADDs (Gomez et al., 2017). Clozapine binds with high affinity to many receptors and has side effects such as behavioral inhibition and potentially fatal agranulocytosis (Magnus et al., 2019). Thus, the use of clozapine as a DREADD actuator in humans may result in undesirable side effects (Gomez et al., 2017). Converted clozapine reaches its maximal concentration at 2–3 h after CNO treatment (Raper et al., 2017), indicating that the effects of CNO on cellular activity are most likely to occur at this time point. These dynamic pharmacological profiles of CNO *in vivo* result in a long and uncontrollable process, which limits the potential for emergency clinical applications such as seizure control (Avaliani et al., 2016).

*Drosophila melanogaster* odorant receptor 35a (OR35a) assembles into heterotetramers with the coreceptor Orco to form a typical ligand-gated cation channel (DasGupta and Waddell, 2008; Nichols and Luetje, 2010). 2-Pentanone is the natural ligand of the channel. In the present study, the designed odorant receptor system (DORs), the complex of OR35a and Orco, was activated by inhalation of 2-pentanone and effectively manipulated physiological processes and rodent behavior on a time scale of minutes. Here, we provide an easy-to-use, minimally invasive, and spatiotemporally controllable approach to manipulate physiological processes. Because of the safety, availability, and cost-effectiveness of 2-pentanone, this “odorgenetic” approach has great potential for clinical therapeutics.

First, we describe our scheme for DOR cloning, design and activation by odorants and the process through which physiological functions are manipulated by this system (Fig. 1A and 1B). The 2-pentanone-induced cation influx triggers intracellular Ca^2+^ increase and membrane depolarization and therefore manipulation of the Ca^2+^- and membrane potential-dependent physiological processes.

**Figure 1.**
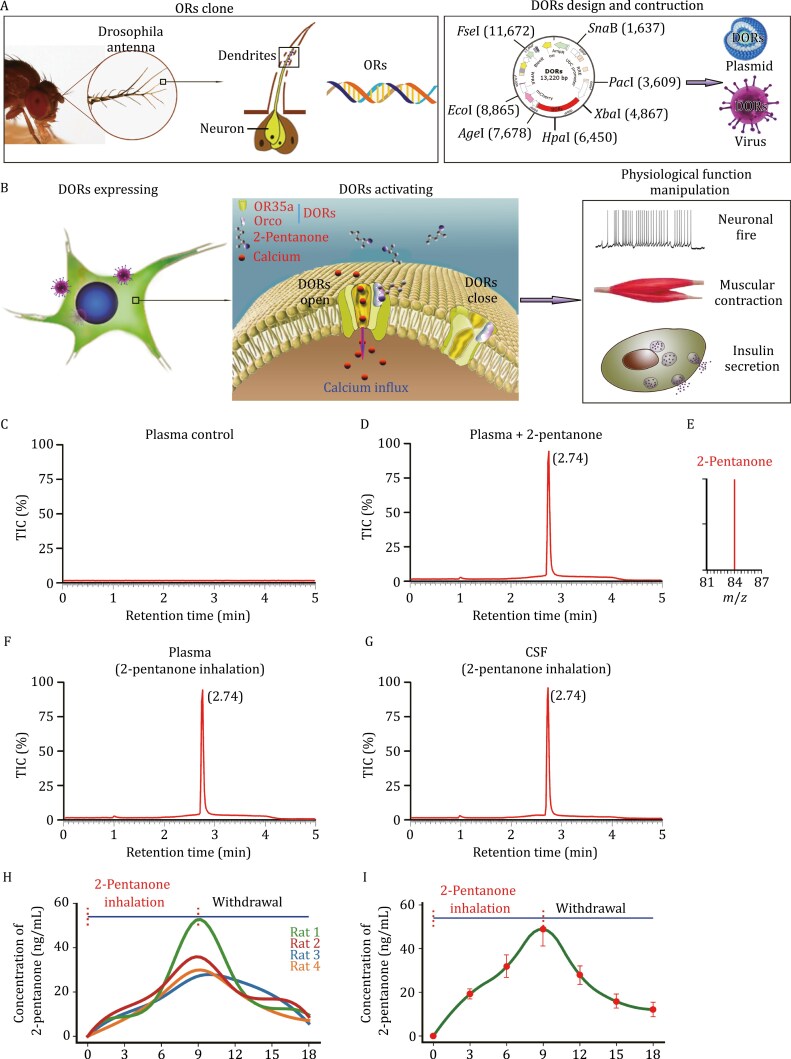
DOR design and manipulator odorant scanning. (A and B) Schematic drawing showing the principle of DOR design and their activation by odorants to manipulate physiological functions. (C and D) Pure plasma and plasma containing 2-pentanone were used as negative and positive controls, respectively, for 2-pentanone examination using LC–MS. According the positive control, 2-pentanone was detectable at the retention time of 2.74 min. (E) 2-pentanone was identified by the mass charge ratio (*m*/*z* = 87). (F and G) The 2-pentanone was detectable in the blood and CSF at 3 min of mice exposed to this compound (2%, *v*/*v*). (H) Dynamic concentration of 2-pentanone in the blood of rats exposed to 2-pentanone (*n* = 4). (I) Dynamic concentration of 2-pentanone in the blood of mice exposed to 2-pentanone showing a rapid time-dependent increase in the concentration of 2-pentanone until the withdrawal of the odorant. Withdrawal of 2-pentanone, the blood concentration of 2-pentanone decreased to approximately 20% of the peak concentration within 10 min (48.88 ± 10.63 ng/mL to 12.11 ± 3.76 ng/mL, *n* = 4 for each time point).

Then, to develop “odorgenetic” approach that is potential for clinical using, we need an odor manipulator that could be unharmful and volatile at room temperature. More than 40 common odorants of food additives were selected (Supplementary Tables). To further test whether the selected odorants enable to deliver into the blood, the odorant in the blood was tested by liquid chromatography‒mass spectrometry (LC–MS). To test the pharmacokinetic characteristics of 2-pentanone, both rats and mice were treated with 2-pentanone inhalation (2%, *v*/*v*). The 2-pentanone concentrations in the blood and cerebrospinal fluid (CSF) was examined using LC–MS. Pure plasma and plasma containing 2-pentanone were used as negative and positive controls, respectively (Fig. 1C and 1D). 2-Pentanone was determined by the mass-to-charge ratio (*m*/*z*: 87, Fig. 1E). The results showed that 2-pentanone enable to be delivered into the blood and then to the CSF quickly by inhalation (Fig. 1F and 1G). The 2-pentanone concentration in the blood of both rats and mice showed a time-dependent increase during inhalation of 2-pentanone and decreased rapidly after withdrawal of the odorant (Fig. 1H and 1I). These results indicated that 2-pentanone had the appropriate profile of spatiotemporally controllable and easy to use to be a candidate manipulator of these DORs.

To verify whether 2-pentanone bound to and opened the DORs on mammalian cells, the results indicated that bath application of 2-pentanone elicited robust calcium influx in DOR-expressing Neuro-2a cells (Fig. 2A and 2B, S1) and HEK293T cells (Fig. S7C–F). Independent expression of either OR35a or Orco could not elicit calcium influx by bath application of 2-pentanone in HEK293T cells (Fig. S7A and S7B). To examine whether 2-pentanone induced dose-dependent neuronal spike of DOR-expression neurons (Fig. S2), the results showed that 2-pentanone elicited robust neuronal spikes in DOR-expressing neurons in a dose-dependent manner (Figs. 2C, 2D, S3, S8, S9, Videos S1 and S2). To test whether DORs can control predatory hunting behaviors by manipulating GABAergic neuronal activity in the CeA, DORs were Cre-dependently expressed in GABAergic neurons in the CeA (Fig S4, Videos S3 and S4), and predatory-like bites induced by inhalation of 2-pentanone were observed (Fig. 2E). The behavioral experiment confirmed that 2-pentanone can manipulate rodent behaviors in a reversible and fast on/off manner (Fig. 2F and Video S5). To verify whether DORs manipulate skeletal muscle in mice, Lentivirus (LVs) expressing DORs were injected into the anterior tibial muscle. The contraction of virus-injected muscles and associated limb movement elicited by 2-pentanone inhalation was observed (Fig. 2G). The results indicated that inhalation of 2-pentanone for a few minutes elicited continuous muscle contraction and limb movements, while withdrawal of the odorant terminated the muscle contraction quickly (Figs. 2H, S5 and Video S6). To test whether DORs can manipulate islet β cells to release insulin (Fig. S6), thereby lowering blood glucose levels, DORs were expressed in the pancreas of mice (Fig. 2I); 2-pentanone inhalation resulted in a significant increase in the blood insulin concentration in pancreatic DOR-expressing mice (Fig. 2J) and therefore lowered the blood glucose level (Fig. 2K). These results indicated that DORs can reversibly manipulate insulin release from pancreatic β cells and therefore decrease blood glucose levels through simple inhalation of 2-pentanone.

**Figure 2.**
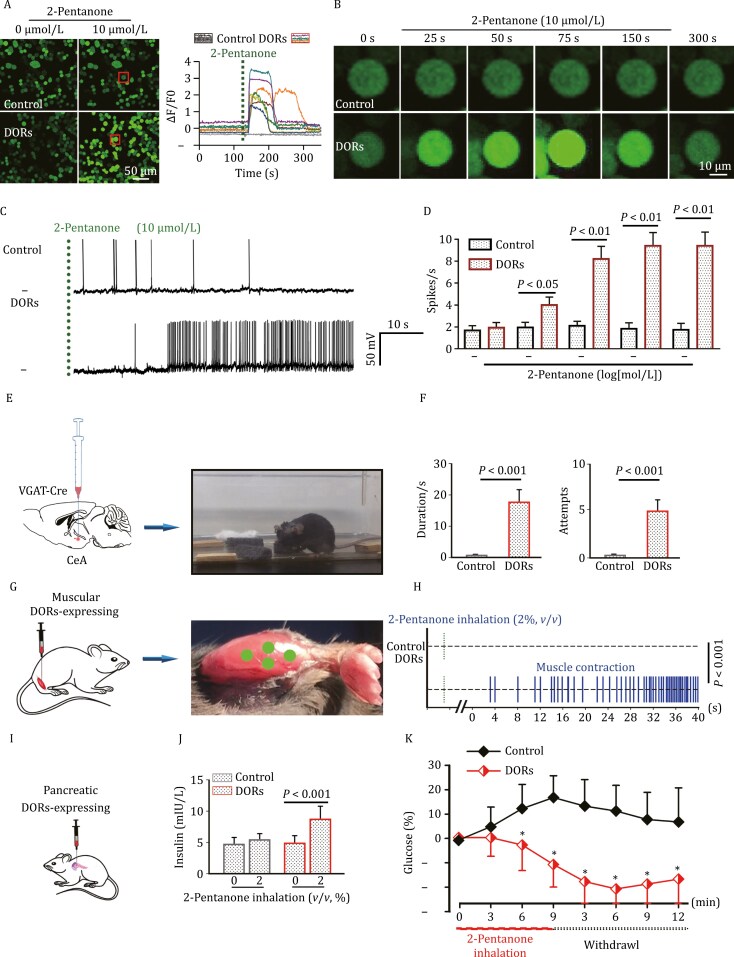
DORs mediate calcium influx, neuronal spiking, control predatory-like behaviors, muscle contraction, and insulin release. (A and B) Two frames (before and after application of 2-pentanone) of time-lapse calcium images of gCaMP coexpressed with DORs or a control in Neuro-2a cells. Time course of fluorescence responses from regions of interest (ROIs), showing a robust calcium influx in DOR-expressing cells in response to 10 µmol/L 2-pentanone. Vertical dashed line indicating treatment of 2-pentanone. (C) Voltage traces showing spikes of DOR-expressing and control-cultured neurons in response to 2-pentanone. Green vertical dashed line showing the time point of 2-pentanone treatment. (D) Compared with the control, 2-pentanone evoked significant spikes in DOR-expressing neurons at different concentrations of 2-pentanone (*n* = 15–20 neurons). Neuronal spikes were counted within 3 min after 2-pentanone treatment. (E) Schematic of virus injection into the CeA of VGAT-Cre mice. (F) Schematic of 2-pentanone inhalation and behavioral observation in freely moving mice. Mean fluorescence intensity change (∆*F*/*F*_0_) of control neurons, DOR-expressing GABAergic neurons, and the inhibited neurons response to 2-pentanone treatment (*n* = 25 of eight slices from five mice). (G) Schematic of virus injection into muscles using a mask in mice. (H) A virus-injected muscle marked by a green oval. Vertical blue lines represent the muscle contraction. (I) Schematic of virus injection into the pancreas. (J) Concentrations of insulin in the blood of mice at 3 min after inhalation of 2-pentanone. (K) Time course of the blood glucose change in mice subjected to 2-pentanone inhalation. Data are shown as the mean ± SEM (*n* = 25, *P < 0.05, repeated-measures ANOVA).

In clinical practice, many diseases are caused by changes in cell activity and function, such as depression, Parkinson’s disease, neocortical seizures, pain, diabetes caused by absolute or relative deficiency of insulin, uterine atony, and heart failure. Manipulating molecular processes governing physiological functions has significant potential for clinical therapeutics of such diseases and is an important approach to elucidate the underlying cellular and physiologic basis. However, for a novel approach using clinical practice, the most important prerequisite conditions are safety, effectiveness, and ease of use. In the present study, we designed an “odorgenetic” system (DORs) to coexpress *Drosophila* odorant receptors consisting of OR35a and Orco and demonstrated that DORs were activated by 2-pentanone and therefore increased intracellular calcium levels by inward rectification. This is the physiological basis of DORs to manipulate calcium-dependent and membrane potential-dependent cellular processes. Then, we demonstrated that DORs enable efficient manipulation of cellular activity and animal behavior, indicating the potential use of DORs in the treatment of various diseases, such as diabetes, Parkinson’s disease, and neocortical seizures (Kätzel et al., 2014).

DORs, as odorant receptors, were exclusively activated by their odor ligand, 2-pentanone. 2-Pentanone is a naturally produced phytochemical that is present in bananas (McCarthy et al., 1964) and carrots (Lan et al., 2013); this colorless liquid ketone has an acetone-like or intensely fruity odor. It is sometimes used in very small amounts as a food additive to impart flavor. 2-Pentanone is soluble in water and volatilizes rapidly to a gas at room temperature. We demonstrated that 2-pentanone enables rapid entry into the blood upon inhalation and leaves the body by exhalation on a time scale of minutes without any significant metabolic process. Thus, using 2-pentanone through the respiratory process to activate DORs in clinical practice has the most important profile: if patients experience manipulation-related adverse events, the manipulation processes can be terminated quickly at any time to maintain patient safety.

Because 2-pentanone volatilizes rapidly to a gas at room temperature, both in basic research and in clinical practice, it is very easy to administer by inhalation to manipulate our DORs *in vivo* without any complex equipment. Thus, DORs overcome many limitations of other methods, including the need for expensive specialized equipment; the difficulty of delivering light to widely distributed cell populations; the invasive procedures required to activate optogenetic systems in deep tissue; and the long, slow pharmacodynamics and irreversible metabolic processes of the designer drugs used in chemogenetics (Becnel et al., 2013; Gomez et al., 2017). DORs are an easy-to-use and spatiotemporally controllable approach to manipulate physiological processes. Because of the safety, availability, and cost-effectiveness of 2-pentanone, this “odorgenetic” approach has great potential for clinical therapeutics. However, if DORs would use in clinic, the odorants receptors expression should be introduced through gene therapy, although the processes of manipulation are reversible upon withdrawal of 2-pentanone, the biosafety of DOR expression in human tissues remains uncertain and further research is needed to identify the impact of DOR expression.

We anticipate that future efforts will be able to develop more “odorgenetic” approaches based on different odorants, and for a functional cellular manipulation system, a series of “odorgenetic” inhibitory approaches is necessary. Using multiple “odorgenetics” with different odorant manipulators, we could manipulate different tissues or different regions of the same organ in an individual with a simultaneous or sequential pattern. These findings would be very beneficial for us to understand the functional relationships between different tissues and organs.

## Supplementary Material

pwae072_suppl_Supplementary_Figures

pwae072_suppl_Supplementary_Table

pwae072_suppl_Supplementary_Video_S1

pwae072_suppl_Supplementary_Video_S2

pwae072_suppl_Supplementary_Video_S3

pwae072_suppl_Supplementary_Video_S4

pwae072_suppl_Supplementary_Video_S5

pwae072_suppl_Supplementary_Video_S6

## Footnotes

This work was supported by the National Natural Science Foundation of China (Original Exploration Program, 32250018 and the National Basic Research Program of China, 81971060). The authors declare no conflicts of interest in this manuscript.
