# Supplementary material for: A minimally invasive, fast on/off “odorgenetic” method to manipulate physiology
Source: Protein Cell. 2024 Dec 31;16(7):615–20. doi: 10.1093/procel/pwae072 (PMC12275085; doi:10.1093/procel/pwae072)
Supplement: pwae072_suppl_Supplementary_Figures [file pwae072_suppl_supplementary_figures.pdf]

Materials And Methods

Animal preparation

Male and female wildtype C57BL/6 (C57) and VGAT-Cre transgenic mice were used to prepare cultured neurons and acute brain slices, and for viral transduction of the cortex, pancreas, and muscle. Animals were maintained in the animal facilities at Hubei University of Medicine in a temperature-controlled animal room with a 12-h/12-h light/dark cycle. All animal procedures were conducted in accordance with institutional guidelines and were approved by the Animal Use and Care Committee of Hubei University of Medicine (Hubei, China). **Examination of 2-pentanone in mouse blood and CSF**

2-Pentanone in blood and CSF was examined by liquid chromatograph mass spectrometry (LC-MS). In brief, mice or rats were anesthetized by an intraperitoneal injection of sodium pentobarbital (50 mg kg $^{-1}$) and then placed in a box with 2% (v/v) 2-pentanone. CSF samples (approximately 20 μl) were collected from the cisternae magnum by a glass pipette with a 50–100-μm-diameter tip. Then, the CSF sample was transferred into a tube with 200 μl acetonitrile and the tube was sealed immediately. Approximately 200 μl of blood was collected from a mouse heart, transferred into a tube with 200 μl EDTA, and sealed immediately. The CSF and blood samples were centrifuged at 1500×*g* for 10 min and the supernatant was harvested for LC-MS analysis. 2-Pentanone was added to 200 μl acetonitrile as a positive control. 2-Pentanone was determined by the retention time and mass charge ratio (m/z). The concentration of 2-pentanone in samples was determined by a standard curve according to total ion

chromatography (TIC).

Plasmid construction

DORs were designed as following: Ubc(promoter)-Or35a-P2A-Orco-P2A for DORs expression in general cell and hSyn(promoter)-LSL(Loxp stop Loxp)-Or35a-P2A-Orco-P2A for Cre-dependent expressing of DORs. The same vector backbone except carrying the mCherry protein was used as negative control. Then, the sequences encoding DORs were cloned into lentivirus vectors. The lentivirus was packaged using three-plasmid transient system.

Cell preparation

HEK293T and Neruo-2a cells were plated on 2×2-mm glass coverslips coated with poly-D-lysine (Sigma-Aldrich). Cells were transfected with plasmids to express DORs and GCaMPs. Then, the cells were imaged with an sp8 confocal microscope (Leica, German) to examine calcium influx elicited by 2-pentanone. DORs-expressing cells were used for patch clamp experiments.

Primary neuronal cultures

Mouse cortical neurons were prepared from postnatal day 0 C57 mouse pups. In brief, cortical neurons were dissociated from dissected P0 mouse brains in 0.25% trypsin-EDTA (GIBCO), plated on 2×2-mm glass coverslips coated with poly-D-lysine, and cultured at 37°C with 5% CO_2 in neurobasal medium (GIBCO) containing 2% B-27 supplement. On day 2, the cultures were coinfected with recombinant adeno-associated viruses to express GCaMPs (AAV-GCaMPs) and lentivirus to express DORs (LV-DORs). On day 5, the cells were imaged by confocal microscopy to examine

calcium influx elicited by 2-pentanone. Cells infected with LV-DORs were used for electrophysiological examination on day 8.

Calcium influx imaging of acute brain slices

AAV-GCaMPs and LV-DORs were injected into the cortex of adult C57BL/6 or VGAT-Cre mice. Two weeks later, brain tissue including the virus injection site was cut into slices (200-μm-thick) with a VT1000S vibratome (Leica) and placed in artificial cerebrospinal fluid for 30 min at room temperature. Slices were imaged by confocal microscopy to examine calcium influx elicited by 2-pentanone.

Artificial cerebrospinal fluid preparation:

Ca^{2+}-fluid :(in mM: 127 NaCl, 25 $NaHCO_3$, 1.25 NaH_2PO_4, 2.5 KCl, 10 Glucose, 2 $MgSO_4$, and 2 $CaCl_2$)

Ca^{2+}-free fluid:(in mM: 129 NaCl, 25 $NaHCO_3$, 1.25 NaH_2PO_4, 2.5 KCl, 10 Glucose, 2 $MgSO_4$)

Electrophysiological recordings

To prepare brain slices, mice were deeply anesthetized and the whole brain was removed and immediately placed to an ice-cold oxygenated sucrose solution (95 mM NaCl, 1.8 mM KCl, 7 mM $MgCl_2$, 0.5 mM $CaCl_2$, 1.2 mM NaH_2PO_4, 25 mM $NaHCO_3$, 11 mM glucose, and 50 mM sucrose; pH 7.4) for sectioning. Mouse brain tissue including the virus injection site was cut into slices (300-μm-thick) using the vibratome and incubated in artificial cerebrospinal fluid for 60 min at room temperature. Whole cell patch clamp recordings were performed with a Multiclamp 700B (Molecular Devices). Pipettes were filled with an internal solution (in mM:135 potassium gluconate, 0.5 $CaCl_2$, 2 $MgCl_2$, 10

EGTA, 10 HEPES, 0.5 Na_3GTP, and 5 ATP-Mg). After obtaining giga-ohm seals, evoked action potentials were elicited in a series of step currents in 20 pA increments from 0–200 pA to test neuronal activity. Then, action potentials evoked by 2-pentanone were recorded. For cultured Neruo-2a cells, the membrane potential was recorded by the current clamp.

Virus injection into muscle and pancreas

For virus injection into muscle and pancreas, mice were anesthetized by an i.p. injection of pentobarbital (50 mg/kg). A procedure was performed to expose the tibialis anterior muscle or pancreas. Then, 1 μl LV-DORs was injected into the tibialis anterior muscle or pancreas using a Nanoliter 2000 Injector (WPI) microsyringe pump and then the surgical incision was closed with a silk suture.

Muscle contraction test

To observe muscle contraction induced by 2-pentanone, mice were anesthetized by injection of pentobarbital (50 mg/kg, i.p.) and the tibialis anterior muscle was exposed. Muscle contraction and limb movement were observed under an SZX16 stereomicroscope (Olympus) and recorded using a camara during inhalation of 2-pentanone.

To test the contraction of virus-injected uterine smooth muscle induced by bath application of 2-pentanone, one side of the uterine was separated and fix on the pressure transducer, and the other side of the uterine was fix with a silk thread to the bottom of the container. The contraction of the uterine smooth muscle was recorded by a was examined by the *in vitro* muscle tone measurement system.

Insulin measurement

Mice with pancreatic DORs expression were anesthetized with pentobarbital (50 mg/kg, i.p.). After 6 min of 2-pentanone inhalation, blood was collected in heparin-containing tubes and centrifugated for 30 min at 1000×*g* at 2–8°C. The insulin concentration was measured using a Mouse INS ELISA kit (BIM) in accordance with the manufacturer's protocol. The sample concentration was determined by a standard curve.

Glucose test

Mice with pancreatic DORs expression were anesthetized with pentobarbital (50 mg/kg, i.p.). During 2-pentanone inhalation, blood glucose from tail vein blood was tested using portable blood glucose meter every 3 minutes. Glucose change showed as percentage= (Glucose(T)- Glucose(0))/ Glucose(0)*100%; Glucose(T): glucose of each time point, Glucose(0): glucose before inhalation of 2-pentanone

Stereotaxic virus injection, calcium influx imaging in vivo, and behavioral assessment

Mice were anesthetized by pentobarbital (50 mg/kg, i.p.) and then placed in a stereotaxic frame for virus injection using the Nanoliter 2000 Injector microsyringe pump. Approximately 3×3 mm of skull was removed and 1 µl LV-DORs and 0.5 µl AAVs-GCaMPs were injected into the cortex (S1: AP: -0.5 mm, ML: 2.5 mm, DV: 0.3 mm, CeA : AP: -1.1 mm, ML: 2.6 mm, and DV: 4.2 mm). After virus injection, the skull window was covered with a glass coverslip fixed by dental cement. The virus was allowed to incubate for 2 weeks. For calcium influx imaging in vivo, mice were anesthetized with pentobarbital (50 mg/kg, i.p.) and placed on the stage of the

confocal microscope. Inhalation of 2-pentanone was carried out with a mask and calcium influx was imaged by the confocal microscope equipped with a 25/0.95 NA water-dipping objective and mode-locked Ti:Sapphire laser (Mai tai) at 910 nm. Images were acquired at 1–2 Hz with a view field of 512 × 512 pixels. Approximately 20 minutes of spontaneous calcium activities were recorded during 2-pentanone inhalation. To observe predatory-like bites, mice were place in a transparent box for inhalation of 2-pentanone (2%, v/v). Hunting behaviors were recorded by a camera.

Supplementary Figures and Figure Legends

S1

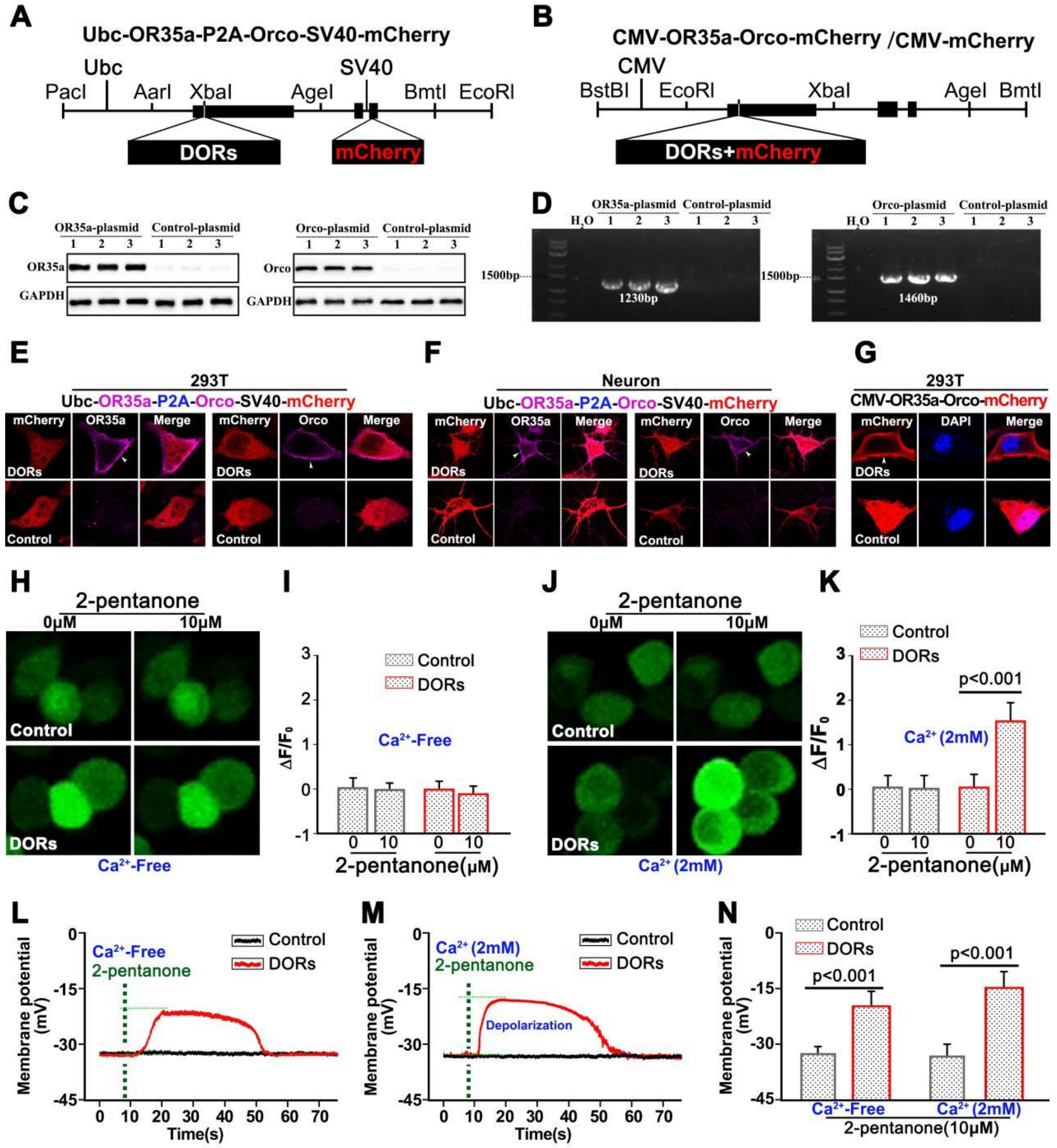

Fig. Legend. S1. DORs mediate calcium-dependent inward calcium influx

(A) Schematic diagrams showing the strategy of insertion site, reporter and linker of DORs expression.

(B) Schematic diagrams showing the strategy of OR expression independence.

(C and D) Three replications of western blot and PCR examination of OR35a and Orco expression, respectively, in HEK293T cells.

(E and F) Immunofluorescence histochemistry showing membrane expression (white

arrowhead) of OR35a and Orco in DOR-expressing HEK293T cells and cultured neurons.

(G). Fluorescence image of mCherry to show the membrane expression (white arrowhead) of DORs directly in HEK293T cells transfected with the plasmid of OR35a and Orco tandem expression following an mCherry reporter.

(H) Two frames (before and after application of 2-pentanone) of time-lapse calcium images of DOR-expressing and control cells in response to 2-pentanone in Ca^{2+}-free solution.

(I) Mean fluorescence intensity change ($\Delta F/F_0$) of DOR-expressing and control cells in response to 2-pentanone in Ca^{2+}-free solution (n=22 cells from four cultures, $p>0.05$).

(J) Two frames (before and after application of 2-pentanone) of time-lapse calcium images of DOR-expressing and control cells in response to 2-pentanone in Ca^{2+}-containing solution.

(K) Mean fluorescence intensity change ($\Delta F/F_0$) of DOR-expressing and control cells in response to 2-pentanone in Ca^{2+}-containing solution (n=20 cells from four cultures, $p<0.001$).

(L-N) Time course recording of the membrane potential of DOR-expressing and control Neuro-2a cells exposed to Ca^{2+}-free solution (L) and Ca^{2+}-containing solution (M) in response to 2-pentanone. (N) Quantitative analyses of the membrane potential of DOR-expressing and control cells after bath application of 2-pentanone in Ca^{2+}-free and Ca^{2+}-containing solution (Ca^{2+}-free solution: mean change =14.45±7.33

mV, $p<0.001$, $n=23$ cells from five culture, Ca^{2+}-containing: mean change =16.38±7.79 mV, $p<0.001$, $n=25$ cells from five cultures).

S1. 2-Pentanone induces calcium influx in DOR-expressing cells

To express OR35a and Orco in a nonspecific manner *in vitro*, OR35a and Orco were separated by a P2A sequence and cloned and inserted into a plasmid with a Ubc promoter and an mCherry reporter (Fig. S1.A). The expression of OR35a and Orco in HEK293T cells was examined by western blotting and PCR, respectively. Three separate bands of western blot and agarose gel of PCR confirmed that both OR35a and Orco were fully expressed by the plasmid (Fig. S1.C and D). Since the membrane location of DORs is a functional prerequisite of DORs, the membrane expression of DORs was verified using immunofluorescence histochemistry with antibodies against OR35a and Orco in DOR-expressing HEK293T cells and cultured neurons transfected using lentivirus. Confocal imaging of HEK293T cells (Fig. S1.E) and cultured neurons (Fig. S1.F) confirmed the membrane location of both OR35a and Orco. To further observe the membrane expression of DORs directly, OR35a and Orco were tandemly expressed following an mCherry reporter in HEK293T cells using a plasmid with a CMV promoter (Fig. S1.B). The fluorescence image of mCherry further confirmed the membrane location of DORs expression (Fig. S1.G).

To verify whether 2-pentanone bound to and opened the DORs on mammalian cells, a gCaMP-expressing plasmid and a DOR-expressing plasmid were cotransfected into HEK293T cells. The time course of fluorescence responses was examined using

confocal microscopy to show that DORs mediated calcium influx by 2-pentanone. The results indicated that bath application of 2-pentanone elicited robust calcium influx in HEK293T cells (Fig. S7.C-F). Independent expression of either OR35a or Orco could not elicit calcium influx by bath application of 2-pentanone in HEK293T cells (Fig. S7.A and B).

The complex of OR35a and Orco is a cation channel. To test whether DOR-mediated calcium influx was extracellularly calcium dependent, HEK293T cells with DOR expression were bathed with calcium-free and calcium-containing solutions during the recording of the calcium response. The results indicated that the DOR-triggered calcium response was extracellular calcium dependent (Fig. S1.H-K). Transmembrane cation influx would change the membrane potential. To test whether DOR induced membrane depolarization was calcium dependent, a whole-cell patch-clamp experiment was performed on DOR-expressing Neuro-2a cells bathing with calcium-free and calcium-containing solutions, and the membrane potential was recorded using current clamp recording. The results showed that 2-pentanone induced significant depolarization of the membrane potential via DORs both in calcium-free and calcium-containing solution. These results confirmed that DORs mediated membrane depolarization was extracellular cations dependent, but not only calcium dependent. (Fig. S1.L-N). These results indicated that DORs enable modulate calcium-dependent and membrane potential-dependent cellular processes, such as neuronal spikes, calcium-triggered endocrine cell secretion, calcium-triggered muscle cell contraction and calcium-triggered immune cell release of toxins.

S2

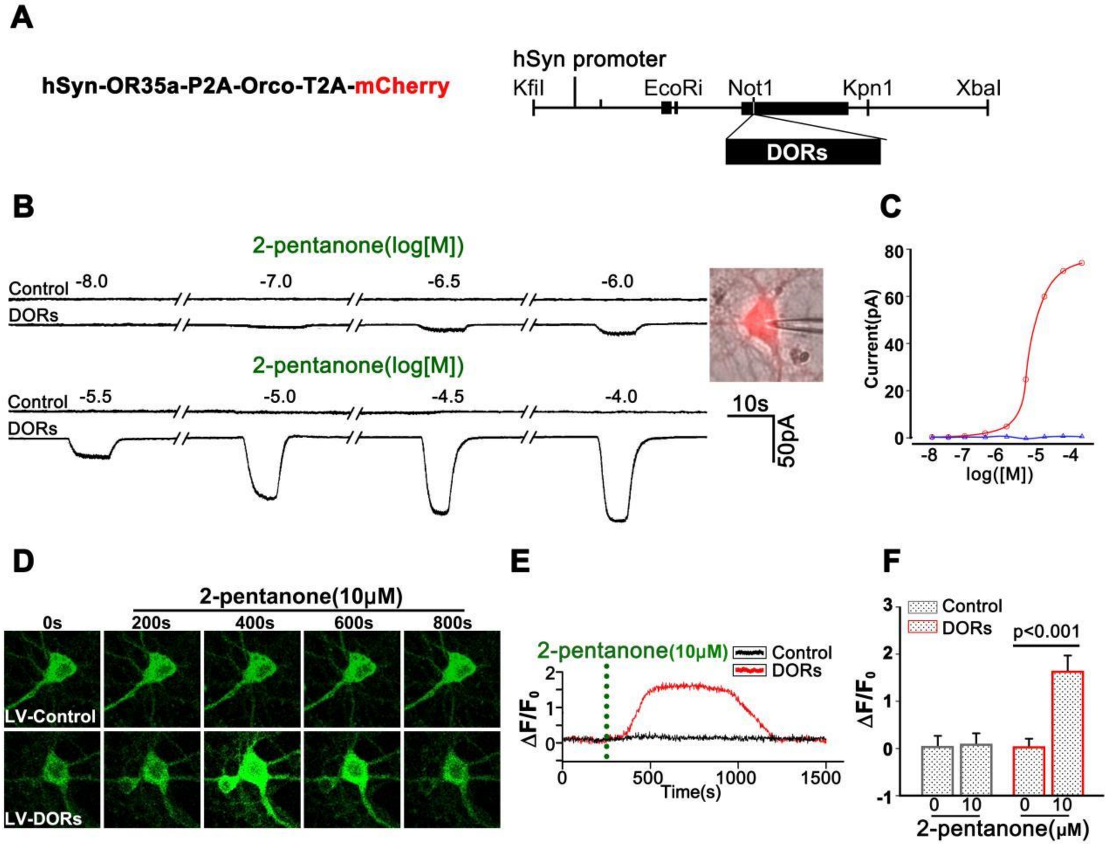

Fig. Legend S2. DORs enable 2-pentanone-driven neuronal spiking *in vitro*

(**A**) Schematic diagrams showing the strategy of DORs neuronal expression.

(B) Continuous recording of transmembrane currents induced by gradient concentrations of 2-pentanone in DOR-expressing and control neurons.

(C) Current response of DOR-expressing neurons to the application of gradient concentrations of 2-pentanone (*n*=18 from four cultures).

(D) Time-lapse calcium images of DOR-expressing and control neurons in response to 2-pentanone.

(E) Time course fluorescence responses of the above neurons in response to 2-pentanone. Green vertical dashed line indicating the timepoint of 2-pentanone

treatment.

(F) Mean fluorescence intensity change ($\Delta F/F_0$) of DOR-expressing and control neurons in response to 2-pentanone (n=24 from four cultures, $p<0.001$).

S2. DOR activation elicits spikes in DOR-expressing neurons *in vivo*

To investigate whether DORs could induce neuronal activity in mice, LVs encoding DORs were injected into the S1 cortex of C57 mice. Then, neuronal spikes of brain slices were recorded using a patch clamp. Quantity analysis of spikes were performed within 3min after 2-pentanone treatment. The results indicated that 10 μM 2-pentanone elicited robust neuronal spikes in DOR-expressing brain slices (Fig. S2.A and B). To examine the DOR-mediated calcium response in DOR-expressing neurons in the brains of mice, LVs encoding DORs and AAVs encoding gCaMP were injected into the S1 cortex of C57 mice. The fluorescence response of brain slices to 2-pentanone was examined. The results showed that bath application of 2-pentanone elicited a continuous fluorescence response in DOR-expressing neurons of brain slices (Fig. S2.C-F and Supplementary Video 1). To further investigate whether DORs can manipulate neuronal activity *in vivo*, LVs encoding DORs and AAVs encoding gCaMP were injected into the S1 cortex of C57 mice, and the neuronal fluorescence response in the cortex of mice during inhalation of 2-pentanone was tested using *in vivo* two-photon calcium imaging (Supplementary Fig. S9.A. Calcium imaging in the S1 cortex *in vivo* showed that a few minutes of 2-pentanone inhalation elicited continuous neuronal activation until 2-pentanone withdrawal (Supplementary Fig. S9.B-D and Supplementary Video 2).

These results confirmed that DORs enabled reversible manipulation of neuronal activation *in vivo*.

S3

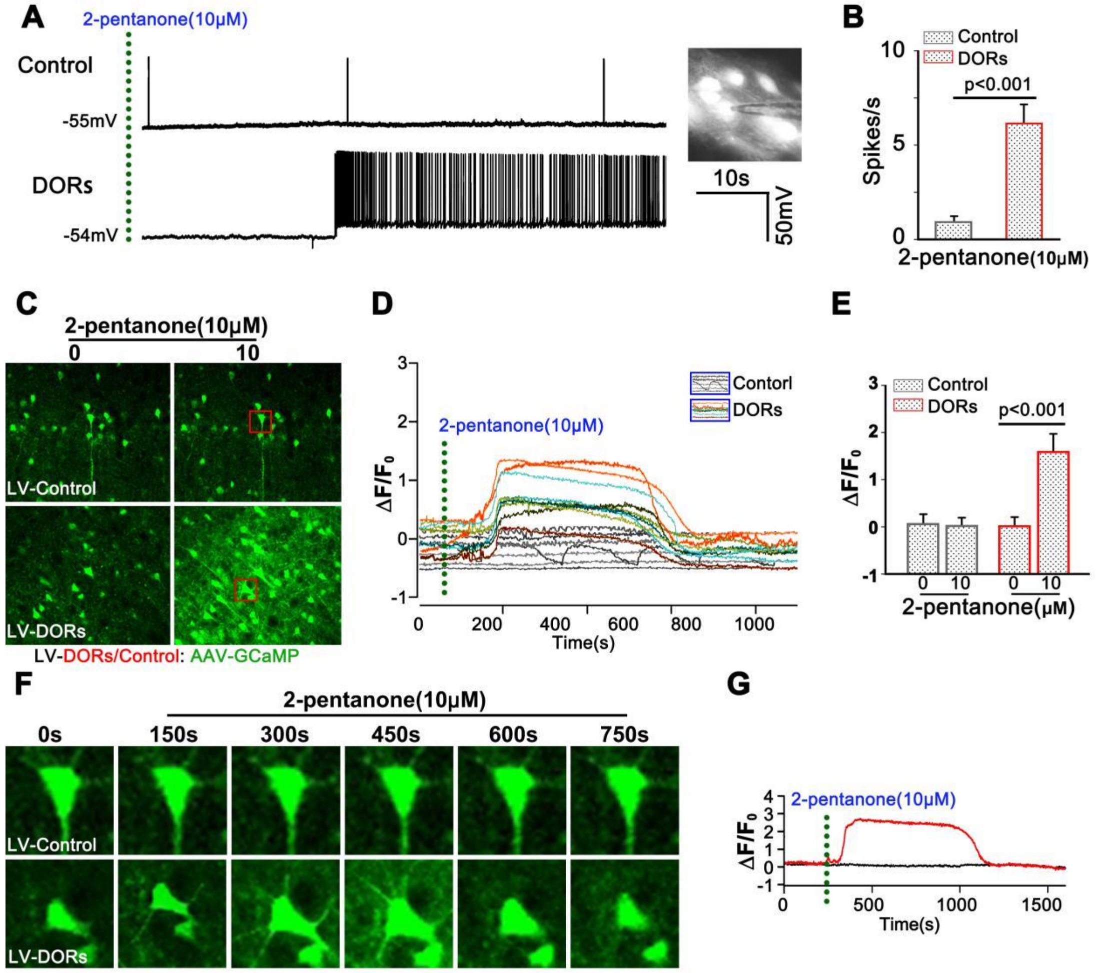

Fig. Legend S3. DORs enable 2-pentanone-driven neuronal spiking *in vivo*

(A) Voltage traces showing spikes of DOR-expressing and control brain slices after bath application of 2-pentanone. The right grayscale photo indicates DORs expression identified by the fluorescence of mCherry during patch clamp experiments. Green vertical dashed line showing the timepoint of 2-pentanone treatment.

(B) Spikes in current-clamped neurons in brain slices within 3min of 2-pentanone treatment (Control, 1.1±0.6 Hz, DORs, 6.9±3.1 Hz, p<0.001, n=36 neurons of nine slices from four mice).

(C) Two frames (before and after application of 2-pentanone) of calcium images in brain slices from C57 mice coinfected with AAV-gCaMP and LVs-DORs or LVs-control.

(D) Time course of fluorescence responses from regions of interest (ROIs) showing a robust calcium influx in DOR-expressing brain slices in response to 2-pentanone. Green vertical dashed line showing the timepoint of 2-pentanone treatment.

(E) Mean fluorescence intensity change ($\Delta F/F_0$) of DOR-expressing and control brain slices (n=16 of six slices from four mice, p<0.05).

(F) Series of calcium image snapshots of control and DOR-expressing sample neurons (marked with red rectangles in the upper images) of brain slices in response to 2-pentanone.

(G) Time course of fluorescence of the sample neurons of the slices in response to 2-pentanone. Green vertical dashed line showing the timepoint of 2-pentanone treatment.

S3. DOR activation elicits spikes in DOR-expressing neurons *in vivo*

To investigate whether DORs could induce neuronal activity in mice, LVs encoding DORs were injected into the S1 cortex of C57 mice. Then, neuronal spikes of brain slices were recorded using a patch clamp. Quantity analysis of spikes were performed within

3min after 2-pentanone treatment. The results indicated that 10 μM 2-pentanone elicited robust neuronal spikes in DOR-expressing brain slices (Fig. S3.A and B). To examine the DOR-mediated calcium response in DOR-expressing neurons in the brains of mice, LVs encoding DORs and AAVs encoding gCaMP were injected into the S1 cortex of C57 mice. The fluorescence response of brain slices to 2-pentanone was examined. The results showed that bath application of 2-pentanone elicited a continuous fluorescence response in DOR-expressing neurons of brain slices (Fig. S3.C-G and Supplementary Video 1). To further investigate whether DORs can manipulate neuronal activity *in vivo*, LVs encoding DORs and AAVs encoding gCaMP were injected into the S1 cortex of C57 mice, and the neuronal fluorescence response in the cortex of mice during inhalation of 2-pentanone was tested using *in vivo* two-photon calcium imaging (Supplementary Fig. S9.A. Calcium imaging in the S1 cortex *in vivo* showed that a few minutes of 2-pentanone inhalation elicited continuous neuronal activation until 2-pentanone withdrawal (Supplementary Fig. S9.B-D and Supplementary Video 2). These results confirmed that DORs enabled reversible manipulation of neuronal activation *in vivo*.

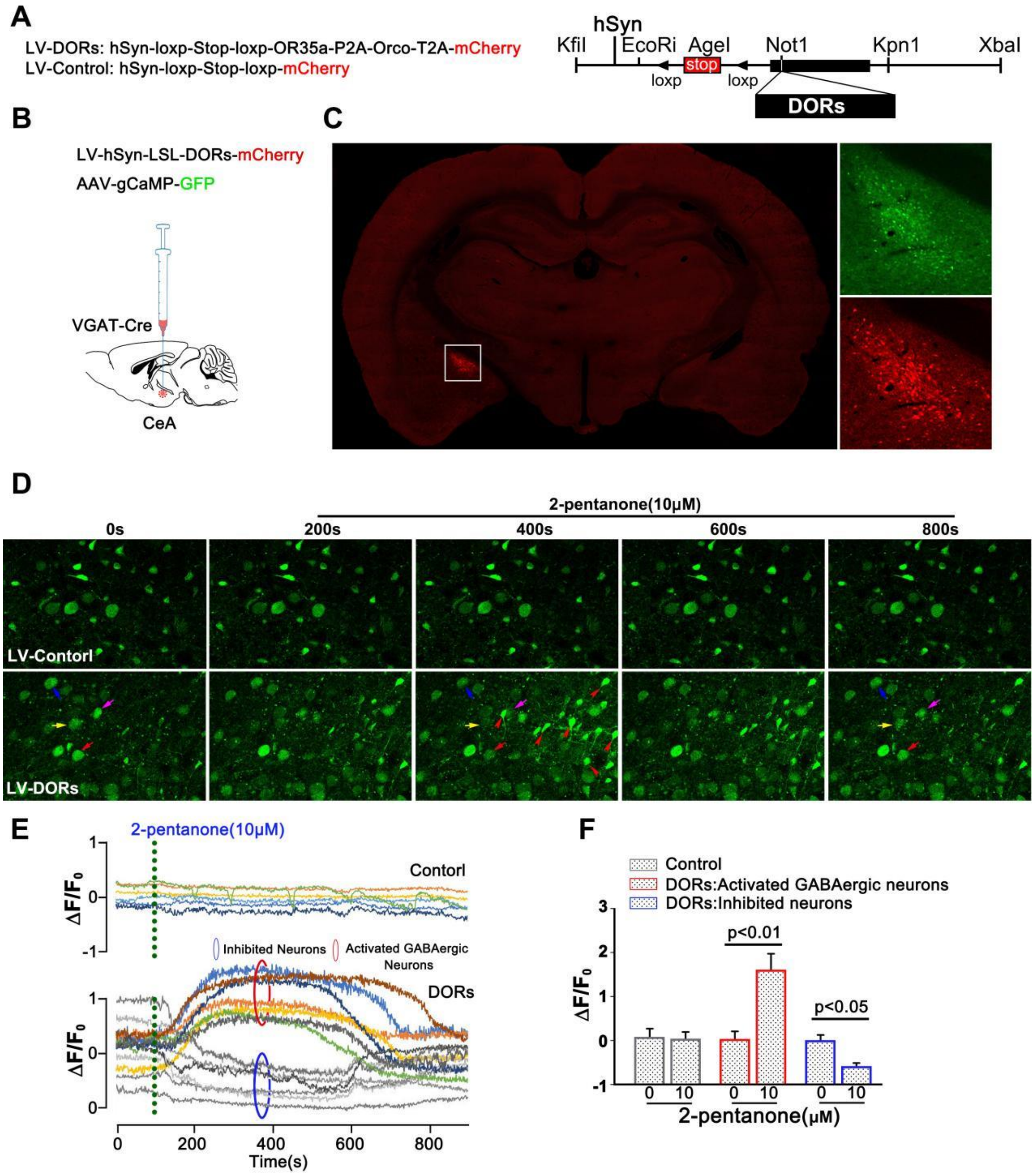

Fig. S4. DORs activate GABAergic neurons in the CeA and control predatory-like behaviors

(A) Schematic diagrams showing the strategy of DORs expression under the control of the Cre-loxp system.

(B) Schematic of virus (LVs encoding loxp-stop-loxp DORs and AAVs encoding gCaMP) injection into the CeA of VGAT-Cre mice.

(C) Whole-brain scanning imaging of mCherry showing DORs expressed in GABAergic neurons in the CeA (marked with white rectangles). The magnified images (shown on the right) indicate both DORs (red) and gCaMPs (green) coexpressed in the CeA.

(D) Time-lapse calcium images of brain slices from VGAT-Cre mice coinfected with AAV-gCaMP and LV-DORs or a control virus in response to 2-pentanone. Red arrowheads indicate activated DOR-expressing GABAergic neurons, and the colorful arrows indicate the downstream neurons that were inhibited by the activated GABAergic neurons.

(E) Time course of fluorescence responses showing that DOR-expressing GABAergic neurons were activated (red oval) and therefore inhibited downstream neurons (blue oval) in response to 2-pentanone. Green vertical dashed line indicating the timepoint of 2-pentanone treatment.

(F) Mean fluorescence intensity change ($\Delta F/F_0$) of control neurons, DOR-expressing GABAergic neurons and the inhibited neurons response to 2-pentanone treatment ($n = 25$ of eight slices from five mice).

Fig. Legend S4. DORs activate GABAergic neurons in the CeA and control predatory-like behaviors

(A) Schematic diagrams showing the strategy of DORs expression under the control of the Cre-loxp system.

(B) Schematic of virus (LVs encoding loxp-stop-loxp DORs and AAVs encoding

gCaMP) injection into the CeA of VGAT-Cre mice.

(C) Whole-brain scanning imaging of mCherry showing DORs expressed in GABAergic neurons in the CeA (marked with white rectangles). The magnified images (shown on the right) indicate both DORs (red) and gCaMPs (green) coexpressed in the CeA.

(D) Time-lapse calcium images of brain slices from VGAT-Cre mice coinfected with AAV-gCaMP and LV-DORs or a control virus in response to 2-pentanone. Red arrowheads indicate activated DOR-expressing GABAergic neurons, and the colorful arrows indicate the downstream neurons that were inhibited by the activated GABAergic neurons.

(E) Time course of fluorescence responses showing that DOR-expressing GABAergic neurons were activated (red oval) and therefore inhibited downstream neurons (blue oval) in response to 2-pentanone. Green vertical dashed line indicating the timepoint of 2-pentanone treatment.

(F) Mean fluorescence intensity change ($\Delta F/F_0$) of control neurons, DOR-expressing GABAergic neurons and the inhibited neurons response to 2-pentanone treatment (n = 25 of eight slices from five mice).

S4. DORs enable fast on/off control of behavior

The central nucleus of the amygdala (CeA) is a modular command system that exerts integrated control of predatory hunting in mice[17], which was reverified through an optogenetic experiment in the present study (Supplementary Video 4). In the duplicated experiments, we reproduced predatory hunting behavior through specific manipulation

of GABAergic neuronal activity in the CeA using optogenetics. To specifically express DORs in GABAergic neurons in the nervous system, DORs were designed to be expressed under the control of the Cre-loxp system. An hSyn promoter following a loxp-stop-loxp sequence was inserted before the DORs sequence, and then the gene sequences were cloned and inserted into a plasmid for lentivirus packaging (Fig. S4.A). To examine whether DORs could selectively manipulate GABAergic neuronal activity, LVs encoding loxp-stop-loxp DORs and AAVs encoding gCaMP were injected into the CeA of VGAT-Cre mice (Fig. 5B). The Cre-dependent expression of DORs was confirmed by the mCherry reporter, and neuronal activity was verified by calcium imaging of brain slices and $\Delta F/F0$ was calculated to show DOR-induced fluorescence response within 3min of 2-pentanone treatment. The results of whole-brain scanning imaging indicated that DORs were expressed exactly in GABAergic neurons in the CeA of VGAT-Cre mice (Fig. 5C). The calcium imaging results showed that 2-pentanone activated the target DOR-expressing GABAergic neurons and therefore inhibited downstream neuronal activity (Fig S4. 5D-F and Supplementary Video 3).The attempts of predatory hunting behaviors were to describe that mouse tries to bite the objective. One times of attempt was to say that the mouse tries to and then takes several bites on the objectives until stop biting. Total time of behaviors observation was 12min including 6min of 2-pentanone inhalation and the following 6min of 2-pentanone withdrawal. Behaviors counting were including the next 3 min of 2-pentanone inhalation and the first 3 min of 2-pentanoen withdrawal. The behavioral experiment confirmed that 2-pentanone can manipulate rodent behaviors in a reversible and fast on/off manner

(Supplementary Video 5). The ability to fast on/off control the activity of specific neurons in freely behaving animals provides an effective way to probe the contributions of neural circuits to behavior and a potential approach for the treatment of neurological diseases in the clinic.

S5

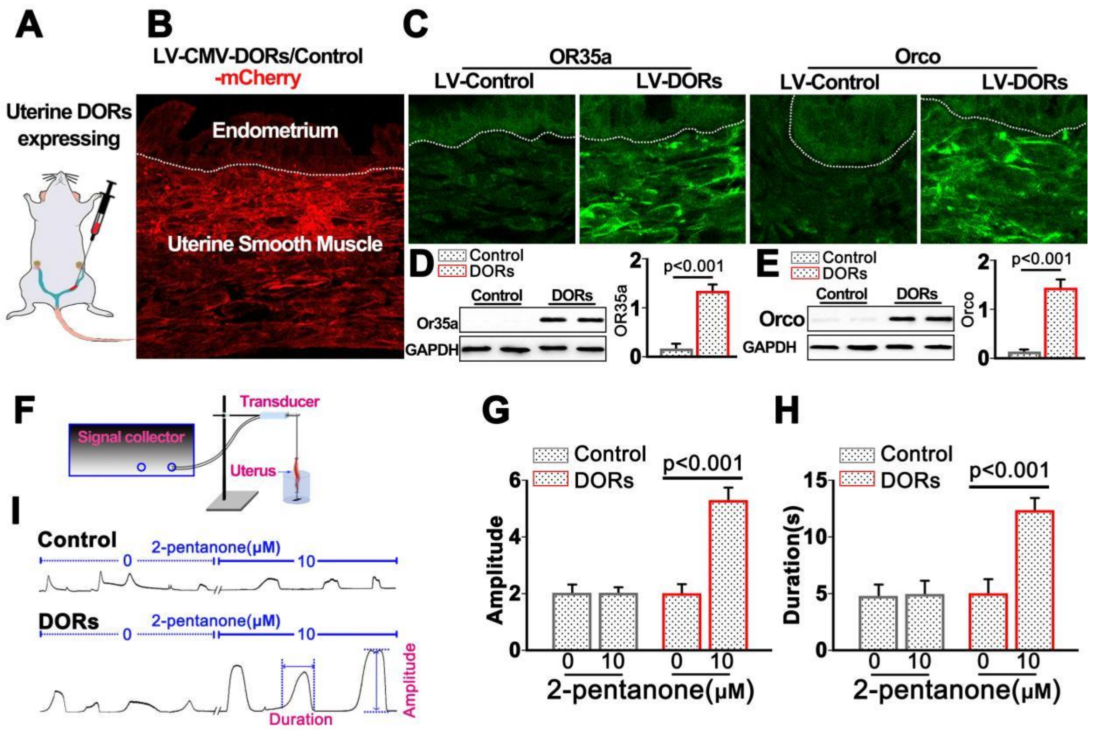

Fig. S5. DORs elicit muscle contraction *in vivo*

(A) Schematic of virus injection into the uterus. Two weeks later, mice were killed, and uterine of virus injection were used to examine DORs expression and test the uterine smooth muscles tension.

(B) Scanning images of mCherry indicating DORs expressed in uterine smooth muscles

(under white dotted line).

(C) Immunofluorescence histochemistry of OR35a and Orco expression in uterine smooth muscle.

(D and E) Two replications of western blot of OR35a and Orco expression in uterine smooth muscle (n= 4, p<0.001).

(F) Schematic of muscle tone measurement *in vitro*.

(G and H) The amplitude and duration time of uterine smooth muscle contraction were analysis in 6 min of bath application of 2-pentanone. Duration time of uterine smooth muscle tension is the time from the beginning to the end of single contraction. Amplitude is the base to the peak of single contraction. Compared with the control, 2-pentanone increased the contraction amplitude and time of uterine smooth muscles expressing DORs. (n= 4, p<0.001, nonparametric Mann–Whitney rank-sum test, two-sided).

(I) Muscle tension curve of DOR-expressing and control uttering smooth muscle in response to 2-pentanone.

S5. DORs enable continuous manipulation of muscle contraction

Skeletal muscle weakness is an important feature of numerous pathological conditions that is linked to impaired locomotion and increased mortality. Muscle contraction is determined predominantly by the excitation–contraction coupling process, which is controlled by intracellular Ca^{2+}. Muscle contraction counting were including the next 3 min of 2-pentanone inhalation and the first 3 min of 2-pentanoen withdrawal.The results indicated that inhalation of 2-pentanone for a few minutes elicited continuous muscle contraction and limb movements, while withdrawal of the

odorant terminated the muscle contraction quickly (Supplementary Video 6).

Uterine atony during cesarean delivery is a serious cause of maternal morbidity and mortality. The generation of uterine smooth muscle contraction is triggered by intracellular Ca^{2+}, while an increase in intracellular Ca^{2+} can promote uterine smooth muscle contraction (1). To test whether DORs could elicit uterine smooth muscle contraction, LVs expressing DORs were injected into the uterus of mice (Fig. 5S.A). The transfection of LVs in uterine smooth muscle was determined by examining the mCherry reporter, and the expression of DORs was examined using immunofluorescence histochemistry and western blotting with antibodies against OR35a and Orco, respectively. The contraction of virus-injected uterine smooth muscle induced by bath application of 2-pentanone was examined by a pressure transducer of an *in vitro* muscle tone measurement system. Immunofluorescence scanning imaging of the reporter in the uterine slice indicated that the uterine smooth muscle was fully transfected with LVs (Fig. 5S.B). The results of immunofluorescence histochemistry staining showed that both OR35a and Orco were fully expressed in the uterine smooth muscle layer (Fig. 5S.C), which was further confirmed by western blotting (Fig. S5.D and E). In the muscle tone measurement experiment, we showed that 2-pentanone reversely enhanced uterine smooth muscle contractility and the duration of each contraction epoch in DOR-expressing uteri (Fig. 6F-I).

S6

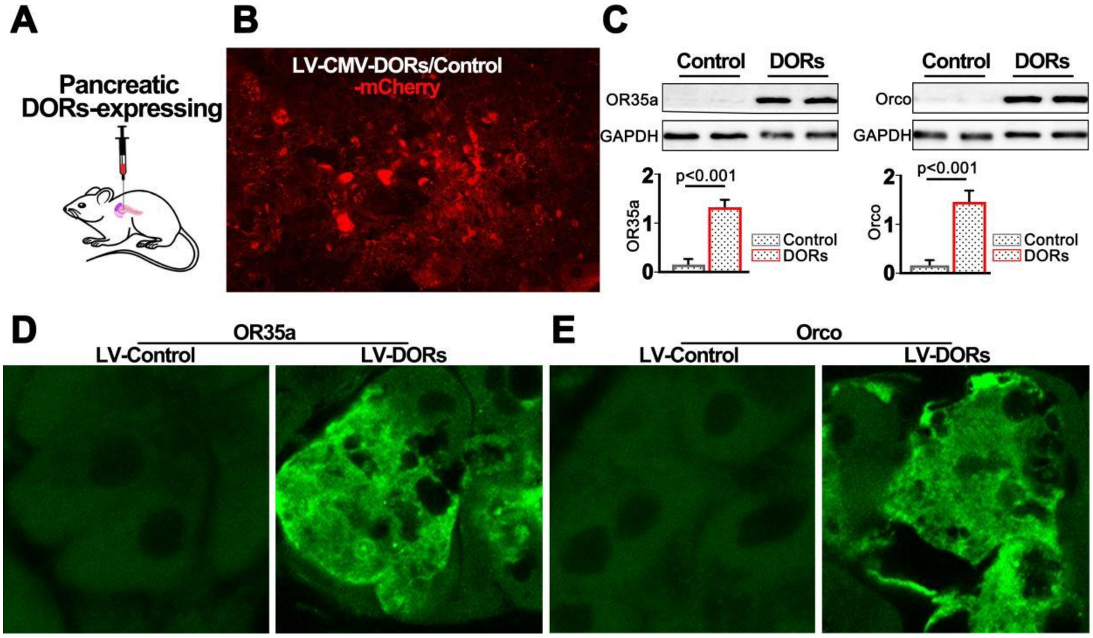

Fig. Legend S6. DORs enable reversible manipulation of insulin release and blood glucose

A) Schematic of virus injection into the pancreas.

(B) Scanning images of mCherry indicating DORs expressed in the pancreas.

(C) Two replications of western blot of OR35a and Orco expression in the pancreas (n= 4, p<0.001).

(D and E) Immunofluorescence histochemistry of OR35a- and Orco-expressing pancreas.

S6. DORs enable reversible manipulation of endocrine activity

Diabetes mellitus is a major worldwide health problem, and its prevalence has been rapidly increasing in the last century. It is caused by defects in insulin secretion, insulin action or both, leading to hyperglycemia. Thus, promoting insulin secretion from pancreatic β cells is an important approach for optimal glycemic control in the clinic. Glucose stimulates insulin secretion at steps distal to the elevation of intracellular

Ca^{2+} in vivo, since DORs enable induction of intracellular Ca^{2+} elevation, to verify whether DORs could trigger insulin release from pancreatic β cells by elevation of intracellular Ca^{2+} and thereby decrease blood glucose levels, LVs expressing DORs were injected into the pancreas of mice (Fig. 7A). The transfection of LVs in the pancreas was determined by examining the reporter of mCherry, and the expression of DORs was examined using immunofluorescence histochemistry and western blotting with antibodies against OR35a and Orco, respectively. Immunofluorescence scanning imaging of the reporter in the pancreatic slice indicated full pancreatic transfection of LVs (Fig. 7B). The results of immunofluorescence histochemistry staining showed that both OR35a and Orco were fully expressed in the pancreas (Fig. 7D and E), which was further confirmed by western blotting (Fig. 7C).

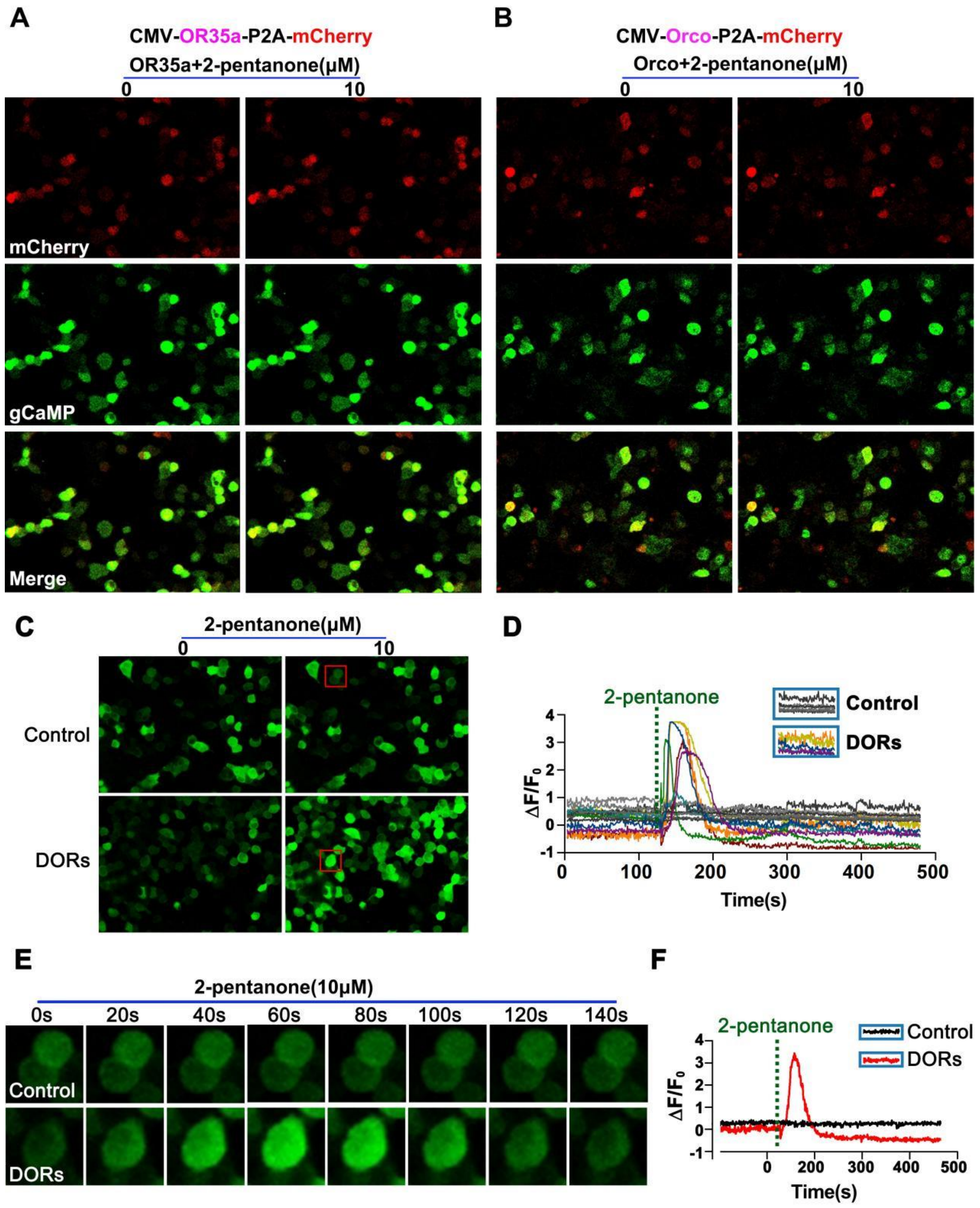

Fig. Legend. S7. DORs induced calcium influx in HEK293T cells

(A and B) Two frames (before and after application of 2-pentanone) of time-lapse calcium images of gCaMP co-expressed with OR35a or Orco respectively and control in HEK293T cells.

(C) Two frames (before and after application of 2-pentanone) of time-lapse calcium

images of gCaMP co-expressed with DORs or a control in HEK293T cells.

(D) Time course of fluorescence responses from regions of interest (ROIs), showing a robust calcium influx in DORs-expressing cells in response to 10 μM 2-pentanone.

(E) Series of calcium images snapshots of the sample cells (marked with red rectangles in up images) in response to 2-pentanone.

(F) Time course fluorescence responses of the sample cells in response to 2-pentanone.

S8

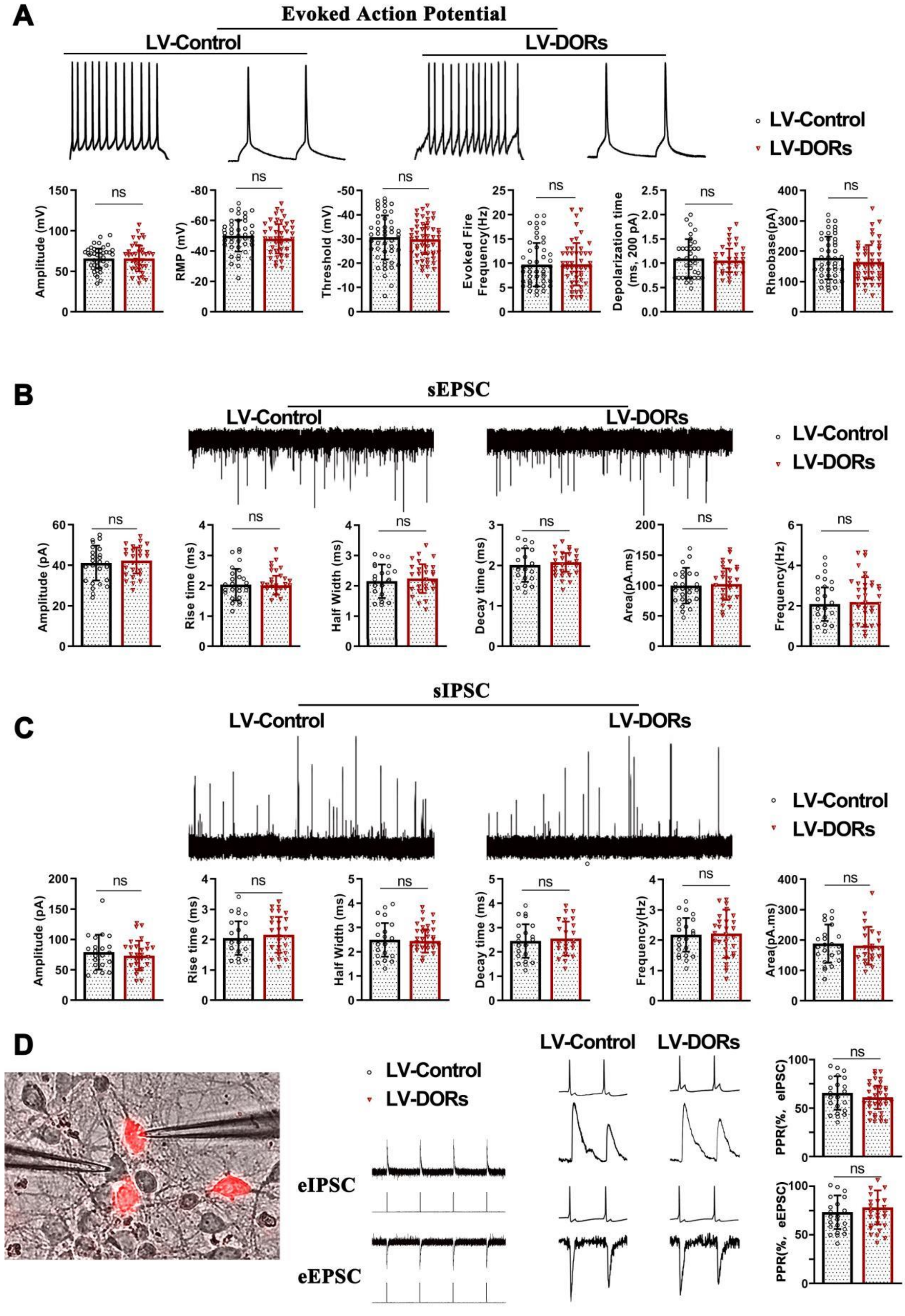

Fig. Legend. S8. DORs expression would not affect neuronal function

(A-D) Whole cell recording of cultured DORs expression neurons which were

identified by the red fluorescence of mCherry during the patch clamp recording. Analyses of spontaneous action potential (A); spontaneous post-synaptic currents (B and C) and evoked post-synaptic current using pair-patch clamp (D), DORs expression would not affect neuronal function of generation of action potential and synaptic transmission.

S9

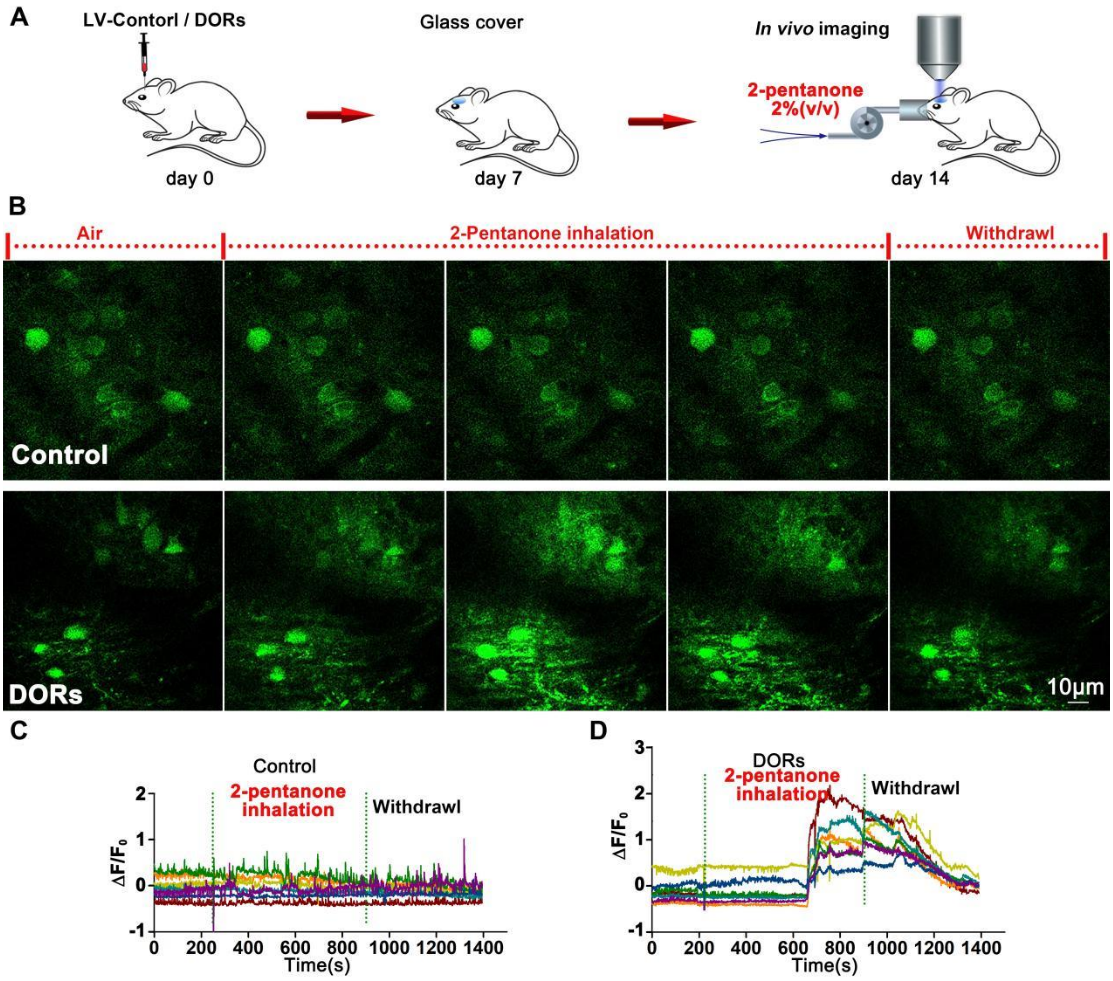

Fig. Legend. S3. DORs elicit neuronal spikes in vivo

(**A**) Schematic of virus injection into the S1 cortex and in vivo calcium imaging in

C57 mice during 2-pentanone inhalation through a mask.

(B) Time-lapse calcium imaging of S1 cortex neurons *in vivo*.

(C,D) Time course of *in vivo* fluorescence responses of control and DOR-expressing neurons in regions of interest (ROIs) in the S1 cortex during inhalation of 2-pentanone. Compared with the control, DOR-expressing neurons showed enhanced fluorescence responses to 2-pentanone inhalation.
